# Identification and adjustment of experimental occlusal interference using functional magnetic resonance imaging

**DOI:** 10.1186/1472-6831-14-124

**Published:** 2014-10-10

**Authors:** Masafumi Oda, Kenichi Yoshino, Tatsurou Tanaka, Shunji Shiiba, Eri Makihara, Ikuya Miyamoto, Shinnosuke Nogami, Shinji Kito, Nao Wakasugi-Sato, Shinobu Matsumoto-Takeda, Shun Nishimura, Keita Murakami, Masahiro Koga, Shigenori Kawagishi, Izumi Yoshioka, Shin-ichi Masumi, Mitsutaka Kimura, Yasuhiro Morimoto

**Affiliations:** Division of Oral and Maxillofacial Radiology, Kyushu Dental University, Kitakyushu, Japan; Department of Oral Health Management, School of Oral Health Sciences, Kyushu Dental University, Kitakyushu, Japan; Division of Dental Anesthesiology, Kyushu Dental University, Kitakyushu, Japan; Division of Occlusion and Maxillofacial Reconstruction, Kyushu Dental University, Kitakyushu, Japan; Division of Oral Medicine, Kyushu Dental University, Kitakyushu, Japan; Division of Oral and Maxillofacial Surgery, Tohoku University Graduate School of Dentistry, Sendai, Japan; Division of Multidisciplinary Studies, Kyushu Dental University, Kitakyushu, Japan; Kyushu Dental University, Kitakyushu, Japan; Center for Oral Biological Research, Kyushu Dental University, Kitakyushu, Japan

**Keywords:** fMRI, Occlusion, Interference, Tooth, Brain, Function, Adjustments

## Abstract

**Background:**

The purpose of this study was to use functional magnetic resonance imaging (fMRI) to quantify changes in brain activity during experimental occlusal interference.

**Methods:**

Fourteen healthy volunteers performed a rhythmical tapping occlusion task with experimental occlusal interference of the right molar tooth at 0 mm (no occlusion), 0.5 mm, and 0.75 mm. The blood-oxygen-level dependent (BOLD) signal was quantified using statistical parametric mapping and compared between rest periods and task periods.

**Results:**

In tapping tasks with experimental occlusal interference of 0.75 mm or 0.5 mm, there was clear activation of the contralateral teeth-related primary sensory cortex and Brodmann’s area 46. At 0 and 30 minutes after removal of the experimental occlusal interference, the activation clearly appeared in the bilateral teeth-related primary sensory cortices and Brodmann’s area 46. At 60 minutes after the removal of the experimental occlusal interference, the activation of Brodmann’s area 46 had disappeared, and only the bilateral teeth-related primary sensory cortices were active.

**Conclusions:**

The present results suggest that adjustments for experimental occlusal interference can be objectively evaluated using fMRI. We expect that this method of evaluating adjustments in occlusal interference, combined with fMRI and the tapping task, could be applied clinically in the future.

## Background

Occlusal information from periodontal mechanoreceptors is used in the control of biting behaviors [1–6]. Adjustment for occlusal interference is necessary in patients with occlusal-related crowns and dental filling restorations. Failure to adjust for occlusal interference may result in compromises in tooth structure, oral mechanics, and quality of life [4–6]. However, the ability to properly adjust for occlusal interference requires a high level of skill from the dentist, because there is no objective consensus on an optimal method of adjusting for occlusal interference. The exact adjustment for slight occlusal interference by the objective evaluations is required.

Recently, studies have investigated the hemodynamic responses observed in the human cortex after dental stimulation [7–18]. Advances in functional brain imaging techniques such as functional magnetic resonance imaging (fMRI) and positron emission tomography allow the cortical representation of dental-related movement or perception to be examined in healthy humans, including the movement and perception of the tongue, lips, and teeth [7–11]. Some studies have used functional brain imaging to study tooth perception [12, 13] and chewing, including parafunction [14–18]; however, to our knowledge, there are no reports on the cortical representation of tooth perception in individuals with occlusal interference. The identification of cortical areas involved in the perception of occlusal interference may offer new methods for occlusion preparation in prosthetic appliances. Therefore, the purpose of this study was to quantify changes in brain activity during experimental occlusal interference.

## Methods

All participants provided written and verbal informed consent to participate in this study prior to undergoing MRI. The institutional review board at Kyushu Dental University approved this study (No. 10–9).

### Subjects

Sixteen healthy right-handed subjects (11 males and five females; mean age, 33.3 years; age range, 25–46 years) with normal masticatory function participated in this study. Normal masticatory function was defined as bilateral biting of food (determined by interview) and the presence of uniform occlusions (determined by examination with occlusal registration paper). None of the subjects had previous fMRI experience. To prevent movement artifacts, subjects rested their head against a flat headrest made of non-magnetic material. Two subjects were excluded from the analysis due to the presence of significant movement artifacts in the imaging data after correction for body movement.

### fMRI parameters

All images were acquired using a 1.5-T full-body MR system (EXCELART Vantage™ Powered by Atlas; Toshiba, Tokyo, Japan) with a circular polarized head coil. Conventional single-section sagittal, coronal, and axial scout images of the head were obtained, and axial and coronal T1-weighted images were obtained for anatomic images with gray/white matter contrast. Functional data were acquired as magnetic susceptibility (T2*)-weighted images with a single-shot gradient echo planar sequence utilizing the blood-oxygen-level dependent (BOLD) technique. The imaging parameters used are shown in Table 1.

**Table 1:** **Imaging parameters**

	Sequences		
	fMRI	T1WI (axial)	T1WI (coronal)
TR (ms)	2000	540	540
TE (ms)	40	15	15
Flip angle (^◦^)	70	70	70
FOV (mm)	250 × 250	230 × 230	230 × 230
Section thickness (mm)	6	3.8	3.8
Echo train spacing	1.2		
Intersection gap (mm)	1	0.2	0.2
Matrix (pixels)	128 × 128	224 × 224	224 × 224

TR: Time of repetition.

TE: Time of echo.

FOV: Field of view.

fMRI: functional magnetic resonance imaging.

T1WI: T1-weighted image.

### Tasks and experimental paradigm

**Figure 1:**
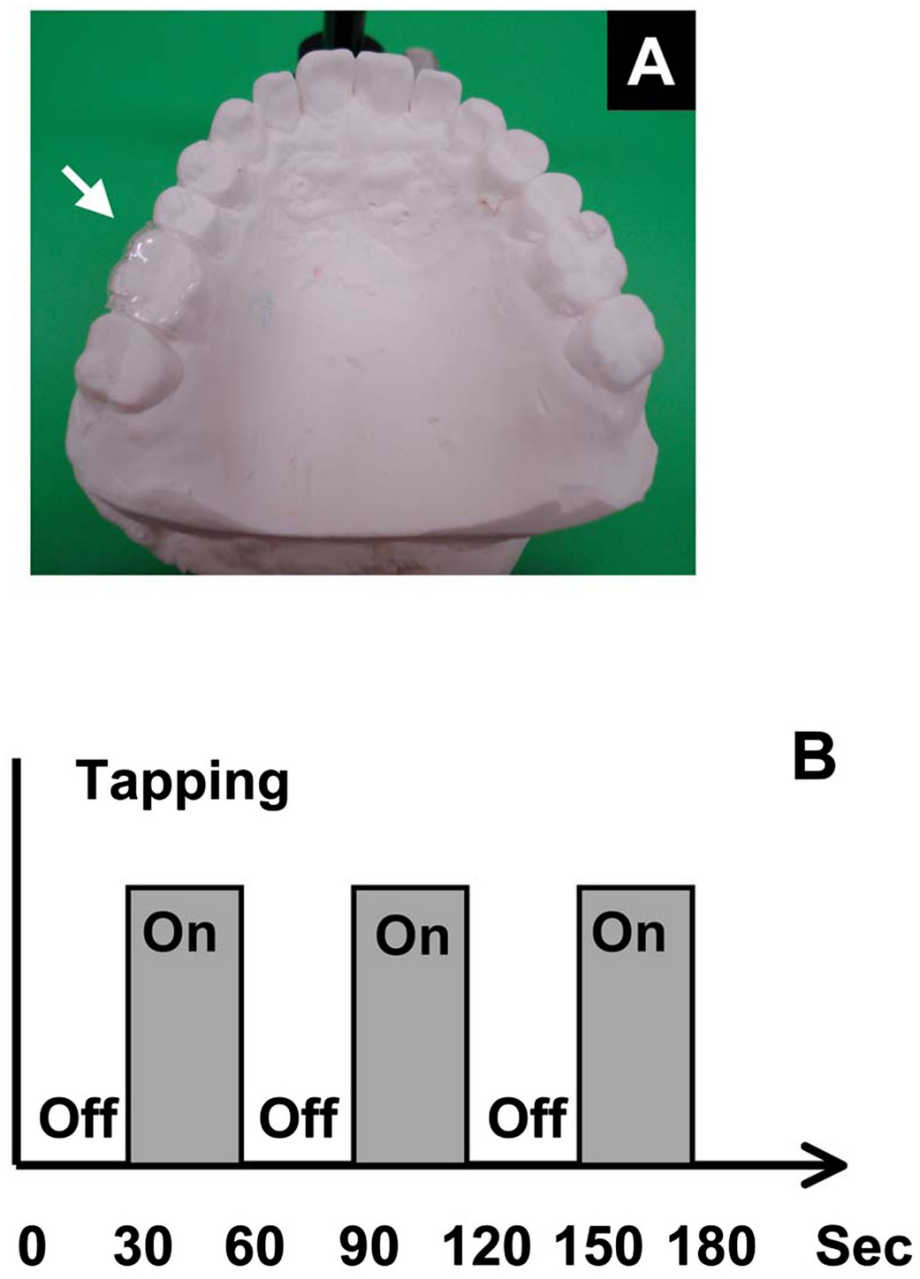
**The device used for experimental occlusal interference and the task paradigm. A)** A photograph of the device used for experimental occlusal interference. **B)** The task paradigm used in the present study.

Subjects performed the task with different levels of experimental occlusal interference in a block paradigm design in which activity periods of 30-s duration were alternated with rest periods of 30-s duration. Experimental occlusal interference was provided by a resin occlusal elevation device (Figure 1A). The occlusal elevation device could provide occlusal interference at three heights: 0 mm (no occlusal interference), 0.5 mm, and 0.75 mm. Subjects performed a rhythmic tapping occlusion task six times: (1) with no occlusal interference, (2) with occlusal interference of 0.75 mm, (3) with occlusal interference of 0.5 mm. This was followed by a repeat of the no occlusal interference condition after a break of 0, 30, and 60 minutes. Subjects remained in the MR scanner while the height of the occlusal elevation device was changed between tasks 1–4. However, subjects did not remain in the MR scanner during the 30-min periods between tasks 4, 5, and 6. In each performance of the task, three successive 60-s cycles were performed (Figure 1B). The task was rhythmic tapping occlusion of the right first molar at a rate of approximately 1 Hz, as described by Onozuka et al. [14]. Before fMRI data acquisition, all subjects were trained to perform rhythmical tapping at a rate of 1 Hz using a metronome. The metronome was not used during fMRI data acquisition to avoid making the subjects anxious. Similarly, bite force was not monitored during fMRI data acquisition to avoid making the subjects anxious.

### fMRI data analysis

Data analysis was performed using SPM 8 (http://www.fil.ion.ucl.ac.uk/spm/software/spm8) executed by Matlab 7.11 (Mathworks, Sherborn, MA, USA). The first five scans of each run were discarded from the analysis due to unsteady magnetization. Differences in slice timing were corrected. The effect of head motion was corrected by realigning all scans to the first scan. After being coregistered with the T1-weighted anatomical volume, images from the functional scans were normalized to the standard Montreal Neurological Institute template.

Thereafter, images were smoothed (8-mm Gaussian kernel) and fitted to hemodynamic response functions. Statistical analysis was performed based on the general linear model approach that observes hemodynamic responses through a linear combination of expected effects up to the level of auto-correlated residual errors [19, 20]. The effects could range from the various tapping task waveforms involved in a hemodynamic response to basis of the rest time in event-related fMRI. Then, subject-specific contrast images of parameter estimates were used for second-level analysis using a random-effects model [20] that accommodated the randomness of differential responses by comparing the mean value in tapping tasks to the variability in tapping tasks from subject to subject to make inferences at a population level. We chose this model because there were random effects on tapping tasks based on the implementation of random effects on tapping tasks analyzed in the context of statistical parametric mapping in normality. A *t* test was used to determine significance on a voxel-by-voxel basis. Areas of activation were characterized by their peak height (*p* < 0.001, uncorrected for multiple comparisons) and spatial extent (>20 voxels).

## Results

### BOLD signal changes associated with rhythmic tapping occlusion

**Figure 2:**
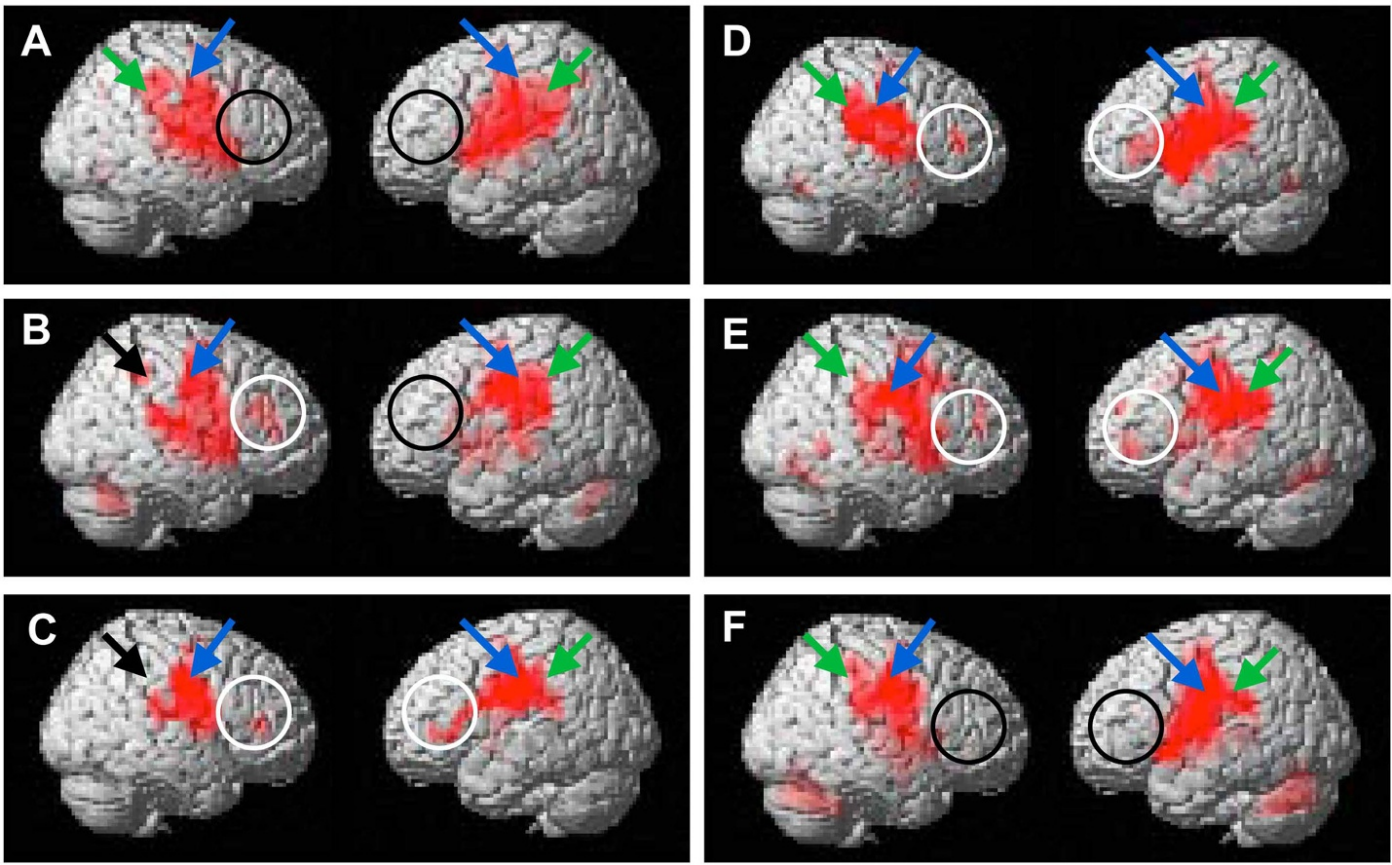
**Surface projection of statistical parametric maps superimposed onto a standard Montreal Neurological Institute template brain (p < 10** ^**-3**^ **) during the tapping task. A)** Performed with no experimental occlusal interference. Bilateral primary sensory cortices (green arrows), the center of the rostral portion of the postcentral gyrus (blue arrows), and Brodmann’s area 46 of the right hemisphere (black circles) were activated. **B)** Performed with 0.75 mm experimental occlusal interference. The contralateral primary sensory cortex (green arrow) and ipsilateral primary sensory cortex (black arrow) and the center of the rostral portion of the postcentral gyrus (blue arrows) were activated. Brodmann’s area 46 in the right (white circle) and left (black circle) hemisphere were activated. **C)** Performed with 0.5 mm experimental occlusal interference. The contralateral primary sensory cortex (green arrow) and ipsilateral primary sensory cortex (black arrow) and the center of the rostral portion of the postcentral gyrus (blue arrows) were activated. Brodmann’s area 46 in both hemispheres (white circles) were activated. **D)** Performed with no experimental occlusal interference immediately after occlusal interference had been removed. Bilateral primary sensory cortex (green arrows) and the center of the rostral portion of the postcentral gyrus (blue arrows) were activated. Brodmann’s area 46 in both hemispheres (white circles) were activated. **E)** Performed with no experimental occlusal interference 30 minutes after occlusal interference had been removed. Bilateral primary sensory cortex (green arrows) and the center of the rostral portion of the postcentral gyrus (blue arrows) were activated. Brodmann’s area 46 in both hemispheres (white circles) were activated. **F)** Performed with no experimental occlusal interference 60 minutes after occlusal interference had been removed. Bilateral primary sensory cortex (green arrows) and the center of the rostral portion of the postcentral gyrus (blue arrows) were activated. Brodmann’s area 46 in both hemispheres (black circles) were not activated.

Firstly, in the tapping task with no occlusal interference, there was a significant increase in the BOLD signal in the bilateral primary sensory cortices (*p* < 0.001; Figure 2A, green arrows) and the center of the rostral portion of the postcentral gyrus (*p* < 0.001; Figure 2A, blue arrows). At the same time, there was a significant increase in the BOLD signal in the bilateral supplementary motor areas (*p* < 0.001), bilateral thalamus (*p* < 0.001), bilateral insula (*p* < 0.001), and bilateral cerebellum (*p* < 0.001; Table 2). However, there was no increase in the BOLD signal in Brodmann’s area 46 in the right hemisphere (Figure 2A, black circles).

**Table 2:** **Neuro-anatomic structures with significant activation during the tapping task**

					Coordinates		
	Region	Side	Broadman area	T values	x	y	z
0 mm	Rostral portion of the postcentral gyrus	R	4	9.55	56	0	34
	Rostral portion of the postcentral gyrus	L	4	14.63	−60	−18	40
	**Primary sensory cortices**	**R**	1-3	9.44	56	−32	46
	Primary sensory cortices	L	1-3	7.94	−60	−38	42
	Supplementary mortor area	R	6	15.29	−8	−6	64
	Supplementary mortor area	L	6	9.94	8	−4	64
	Thalamus	R		8.45	34	0	2
	Thalamus	L		12.63	−32	−4	4
	Insula	R	13	6.94	28	−26	0
	Insula	L	13	8.7	−26	−22	4
	Cerebellum			6.76	0	−68	−10
0.75 mm	Rostral portion of the postcentral gyrus	R	4	13.41	−54	−12	42
	Rostral portion of the postcentral gyrus	L	4	7.76	52	−18	36
	Primary sensory cortices	L	1-3	9.25	−58	−24	42
	**Prefrontal area**	**R**	46	5.42	44	32	18
0.50 mm	Rostral portion of the postcentral gyrus	R	4	7.66	56	−4	28
	Rostral portion of the postcentral gyrus	L	4	14	−56	−4	26
	Primary sensory cortices	L	1-3	8.74	−58	−22	34
	**Prefrontal area**	**R**	46	5.74	40	38	6
	**Prefrontal area**	**L**	46	7.59	−44	28	2
0 mm	Rostral portion of the postcentral gyrus	R	4	10.84	56	0	28
	Rostral portion of the postcentral gyrus	L	4	9.97	−56	−6	20
	**Primary sensory cortices**	**R**	1-3	11.51	−52	−18	22
	Primary sensory cortices	L	1-3	6.9	−62	−22	40
	**Prefrontal area**	**R**	46	6.1	44	42	14
	**Prefrontal area**	**L**	46	5.11	−34	34	6
After 30 min	Rostral portion of the postcentral gyrus	R	4	6.15	62	−8	30
	Rostral portion of the postcentral gyrus	L	4	6.48	−56	−10	28
	**Primary sensory cortices**	**R**	1-3	4.85	56	−36	28
	Primary sensory cortices	L	1-3	10.02	−56	−24	28
	**Prefrontal area**	**R**	46	5.18	34	40	14
	**Prefrontal area**	**L**	46	6.4	−46	44	0
After 60 min	Rostral portion of the postcentral gyrus	R	4	7.56	58	−4	30
	Rostral portion of the postcentral gyrus	L	4	9.5	−54	−10	28
	**Primary sensory cortices**	**R**	1-3	5.59	62	−24	28
	Primary sensory cortices	L	1-3	5.91	−54	−32	26

R: Right.

L: Left.

The locations that BOLD signals changed significantly are highlighted in bold.

### Alterations in local BOLD signal distribution in the primary sensory cortices in occlusal tasks were dependent on the degree of occlusal interference

In tapping tasks with occlusal interference of 0.75 mm, the BOLD signal clearly increased in the left primary sensory cortex (Figure 2B, green arrow), but not in the right primary sensory cortex (Figure 2B, black arrow) or the bilateral center of the rostral portion of the postcentral gyrus (Figure 2B, blue arrows). At the same time, the BOLD signal clearly increased in Brodmann’s area 46 in the right hemisphere (Figure 2B, white circle), but not in the left hemisphere (Figure 2B, black circle). With occlusal interference of 0.5 mm, the BOLD signal clearly increased in the left primary sensory cortex (Figure 2C, green arrow), but not in the right primary sensory cortex (Figure 2C, black arrow). However, the BOLD signal clearly increased bilaterally in the center of the rostral portion of the postcentral gyrus (Figure 2C, blue arrows) and Brodmann’s area 46 (Figure 2C, white circles). In the subsequent tapping task with no occlusal interference, the amplitude of the BOLD signal in the cortical somatosensory regions became bilateral (Figure 2D, green arrows), and bilateral activation of Brodmann’s area 46 remained (Figure 2D, white circles).

As in the task, the BOLD signal clearly increased in the bilateral supplementary motor areas, bilateral thalamus, bilateral insula, bilateral cerebellum, and bilateral prefrontal areas (Table 2). The locations of the clearest foci of activation for these regions are summarized in Table 2 (anatomical regions with maximal *t* values in clusters and the Montreal Neurological Institute coordinates).

### The maintenance and disappearance of local BOLD signal distributions in the primary sensory cortices in occlusal tasks performed after experimental occlusal interference

In tapping tasks performed 0 and 30 minutes after the removal of occlusal interference, the BOLD signal clearly diffusely and bilaterally increased in Brodmann’s area 46 (Figure 2E, white circles) and the cortical somatosensory representation (Figure 2E, green arrows). However, in the tapping task performed 60 minutes after the removal of occlusal interference, the BOLD signal was no longer present in Brodmann’s area 46 in either hemisphere (Figure 2F, black circles).

## Discussion

One of the important results of the present study is that the BOLD signal in the left cortical somatosensory region was increased during a molar-tapping task performed with experimental occlusal interference of the right first molar.

Penfield and Rasmussen intraoperatively investigated human sensory somatotopy [21] and reported that the teeth, gingiva, and jaw were represented in the cortical somatosensory representation. Converging results from magnetoencephalography [22] and tactile stimulation [23] studies indicate that the sensory representation of the oral area is located in the primary somatosensory cortex, the so-called ‘sensory homunculus’. The left superior frontal gyrus was activated during unilateral chewing on the right side [15]. Therefore, we speculated that activation of the left somatosensory cortex would be affected by experimental occlusal interference of the right first molar, and that this would be captured by fMRI. Our results indicate that experimental occlusal interference can be objectively visualized by fMRI.

Activation changed from the contralateral somatosensory cortex to the bilateral somatosensory cortices after the removal of the experimental occlusal interference. This suggests that the legitimacy of adjustment for the occlusal interference using fMRI was elucidated. To our knowledge, this is the first report of the utility of fMRI for assessing the effect of occlusal interference. We expect that, in the future, this method of assessing the presence of occlusal interference, i.e., fMRI during the tapping task, could be applied clinically. In particular, it may be useful to assess the presence of occlusal interference in patients who cannot judge the occlusion themselves.

Another important result of the present study is that Brodmann’s area 46 was activated during experimental occlusal interference of the right first molar. Activation of this area was not present 60 minutes after the removal of experimental occlusal interference. It has been reported that Brodmann’s area 46 as well as in insula controls higher brain functions including the stress-sensitive neuromodulatory systems, which, in turn, control sympathoadrenal and hypothalamic-pituitary-adrenal activity [24–26]. Therefore, the present data suggest that the experimental occlusal interference was an acute stressor. The present results indicate that the disappearance of occlusal interference can be judged by the disappearance of activation of Brodmann’s area 46 in addition to the development of bilateral activation of the cortical somatosensory regions. The insula also controls higher brain functions including the stress-sensitive neuromodulatory systems, but the alterations of BOLD signals could not be caught according to the experimental interferences in the present study. We could not appropriately explain the reason. The possible explanation was that there might be a subtle distinction about response’s phenomenon between the Brodmann’s area 46 and insula.

To our surprise, activation in Brodmann’s area 46 didn’t disappear immediately after the removal of the experimental occlusal interference, and did not disappear until 60 minutes after this time point. The present result suggests that a patient’s adjustment for occlusal interference should make a better result for some time at least over 1 hour. Therefore, our results could recommend the observation i.e. one day, one week etc. after the adjustment of the occlusal interference in dental office.

In the tapping task used in the present study, there was bilateral and uniformly diffuse activation of the bilateral inferior aspect of the primary motor cortex close to the lateral fissure, and the bilateral insula, bilateral thalamus, and bilateral cerebellum (Table 2), in agreement with previous positron emission tomography [10] and fMRI [14, 15, 27] findings. These regions are believed to receive sensory information from the mandibles and the temporomandibular joint, and to control masticatory movements and the lingual and facial muscles [28, 29]. Based on the conformity between our results and previous reports about activation’s areas during the present task, it would be quite appropriately done.

In the present study we used fMRI to investigate the relation between occlusal interference and brain activity because the low spatial and temporal resolution of positron emission tomography makes it difficult to monitor brain activity during tapping tasks. fMRI allows the activity of precise brain regions to be linked to the performance of the tapping task. We selected the BOLD technique because of both the general knowledge and the perfect establishment of its technique. The BOLD technique permits the depiction of slow-flow vessels in T2-weighted images and the depiction of fast-flow vessels by acquiring images with electrocardiogram triggering during the slow-flow cardiac phase [10–13, 22]. With the experimental occlusal interference technique used in this study, the occlusal height of the right first mandibular molar could be raised by up to 0.75 mm using the whole crown covered with resin. For this reason, occlusion between the maxillary and mandibular first molars should be a key role for individuals with normal occlusion. In addition, the maxillary and mandibular first molars tend to be repaired by dental restorations because they have the earliest eruption of all permanent teeth and the longest existence in the oral cavity.

One possible limitation of our present study is the small sample size. In addition, we included only healthy volunteers of a young age. There may be changes in occlusion with age, and it is not clear if our results can be generalized beyond young adults with normal occlusion. Larger and more varied samples should be studied to establish the generalizability of our results to patient populations with a variety of occlusions. In addition, the present study design was that placement of a restoration in hypo-occlusion or for that matter simply placement of a new restoration that changes buccal, lingual morphology or interproximal contact pressure could result in the same types of activations as seen in the hyper-occlusion state. So, the BOLD signal activations that are observed up to 60 min post-removal of the interference is indicative that brain activation patterns may vary as the conditions in the oral cavity change over the short term. Moreover, fMRI cannot be performed on all patients in the clinic because it requires an MRI system. However, we expect that this method of adjusting occlusal interference, combined with fMRI and the tapping task, could be applied clinically in the future. In particular, it may be useful for patients who cannot judge their exact occlusion themselves. For future work, moreover, we also consider the clinical applications of near-infrared spectroscopy (NIRS) methods for adjusting occlusal interference based on our present results. As NIRS is less prone to movement artifact, allows subjects to be in a sitting position, like fMRI, is less expensive and therefore more likely to be the method of choice in a dental setting.

## Conclusion

In the present study, 14 volunteers underwent fMRI when performing rhythmical tapping occlusions with experimental occlusal interference of the right molar tooth at three occlusal heights, in order to objectively establish the adjustment for occlusal interference by brain activations. The alteration in BOLD signal was quantified using statistical parametric mapping of the comparison between rest periods and task periods. In tapping tasks with experimental occlusal interference of 0.75 mm or 0.5 mm, activation was detected in Brodmann’s area 46 and the contralateral teeth-related primary sensory cortex. Brodmann’s area 46 remained active in tapping tasks without experimental occlusal interference performed immediately and 30 minutes after the experimental occlusal interference was removed, but was not active in a task performed 60 minutes after the experimental occlusal interference was removed. These results suggest that adjustments for occlusal interference can be objectively evaluated using fMRI. We expect that this method of adjusting occlusal interference, combined with fMRI and the tapping task, could be applied clinically in the future.
